# Microarray for serotyping of *Bartonella *species

**DOI:** 10.1186/1471-2180-7-59

**Published:** 2007-06-25

**Authors:** Cyrille J Bonhomme, Claude Nappez, Didier Raoult

**Affiliations:** 1 Unité des Rickettsies, CNRS UMR 6020, Faculté de Médecine de Marseille, 13385 Marseille cedex 05, France

## Abstract

**Background:**

Bacteria of the genus *Bartonella *are responsible for a large variety of human and animal diseases. Serological typing of *Bartonella *is a method that can be used for differentiation and identification of *Bartonella *subspecies.

**Results:**

We have developed a novel multiple antigenic microarray to serotype *Bartonella *strains and to select poly and monoclonal antibodies. It was validated using mouse polyclonal antibodies against 29 *Bartonella *strains. We then tested the microarray for serotyping of *Bartonella *strains and defining the profile of monoclonal antibodies.

*Bartonella *strains gave a strong positive signal and all were correctly identified. Screening of monoclonal antibodies towards the Gro EL protein of *B. clarridgeiae *identified 3 groups of antibodies, which were observed with variable affinities against *Bartonella *strains.

**Conclusion:**

We demonstrated that microarray of spotted bacteria can be a practical tool for serotyping of unidentified strains or species (and also for affinity determination) by polyclonal and monoclonal antibodies. This could be used in research and for identification of bacterial strains.

## Background

Within the last 15 years, several bacteria of the genus *Bartonella *were recognized as zoonotic agents in humans and isolated from various mammalian reservoirs. Pets represent a large reservoir for human infection, including exotic pets, because most *Bartonella spp*. infecting them are zoonotic. Cats are the main reservoir for *Bartonella henselae, B. clarridgeiae*, and *B. koehlerae*. Dogs can be infected with *B. vinsonii subsp. berkhoffii, B. henselae, B. clarridgeiae, B. washoensis, B. elizabethae, and B. quintana *[1,2]. Members of the genus *Bartonella *have historically been connected with human diseases, such as cat-scratch disease, trench fever, and Carrion's disease, and recently, have also been recognized as emerging pathogens causing other clinical manifestations in humans [3]. It has been shown that *Bartonella spp*. alone may cause 3% of all cases of infectious endocarditis. Endocarditis is a life-threatening disease, for which a favourable rapid etiological diagnosis must be made as early as possible [4]. Trench fever, a louse-borne disease caused by *Bartonella quintana*, was reportedly reemerging recently in homeless persons [5,6]. Identification of the many species is based either on isolation of the bacterium or PCR testing; nine *Bartonella *species or subspecies have been recognized as zoonotic agents, including *B. henselae, B. elizabethae, B. grahamii, B. vinsonii subsp. arupensis, B. vinsonii subsp. berkhoffii, B. grahamii, B. washoensis *and more recently *B. koehlerae *and *B. alsatica *[7,8]. Serotyping provides valuable information on the incidence of some bacterial serotypes, such as *Salmonella*, *Pneumococcus*, and *Haemophilus*, for which typing can be performed by the slide agglutination method, multiplexed latex beads or PCR [9-12]. Various microarray methods have been described for DNA, RNA and protein analysis than can be applied in the diagnosis of infectious diseases [13]. These microarray systems allow several tests to be performed simultaneously without separating the original sample [14]. In recent years, monoclonal antibodies have become common tools, which can be used for serological assays to detect drugs, hormones and serum proteins; for diagnosis of viral and bacterial diseases; for tumour identification; and for purification of antibodies, proteins and cells [15-18]. Our goal was to develop a multiple antigenic microarray able to detect and *identify by serology *each of the *Bartonella *strains.

To test the bacterial microarray, we studied a collection of polyclonal antibodies directed against the *Bartonella *strains. We initially produced mouse polyclonal sera against twenty-nine *Bartonella *strains and determined optimal dilutions using an array using *Bartonella*.

Furthermore, to confirm the usefulness of our system for serotyping, we produced a new array containing four *Bartonella *strains responsible for major human diseases, using some bacterial strains as blind test controls and *Bartonella *strains isolated by blood culturing from homeless people during a visit of shelters in Marseilles.

Finally, we used our protein microarray for screening monoclonal antibodies generated against the *gro EL *protein of *B. clarridgeiae*.

## Results

### Production of polyclonal sera

We obtained a polyclonal serum against each strain used in the present study. All sera exhibited titers ranging from 1: 3,200 to 1: 12,800 against their respective immune strain (Table 2). Therefore, we decided to test two dilutions for each polyclonal serum: 1:100 and 1:1,000 and in case of a strong cross-reaction, 1: 1,000 and 1:5,000 on slides with spotted array.

**Table 2:** Mouse polyclonal antibodies and titers

Species	Titers
*Bartonella alsatica*	6400
*Bartonella bacilliformis*	12800
*Bartonella bovis*	6400
*Bartonella clarridgeiae*	6400
*Bartonella elizabethae*	12800
*Bartonella henselae *Houston	6400
*Bartonella henselae *Marseille	3200
*Bartonella koehlerae*	3200
*Bartonella quintana *Oklahoma	6400
*Bartonella rattimassiliensis*	6400
*Bartonella tribochorum*	3200
*Bartonella vinsonii arupensis*	12800
*Bartonella vinsonii berkhoffii*	6400
*Bartonella birtlesii*	6400
*Bartonella capreoli*	12800
*Bartonella chomelii*	12800
*Bartonella doshiae*	6400
*Bartonella grahamii*	12800
*Bartonella taylorii*	6400
*Bartonella schoenbuchensis*	12800
*Bartonella vinsonii vinsonii*	3200
*Bartonella phoceensis*	12800
*Bartonella *from *Macropus giganteus *(Australia 1)	6400
*Bartonella *from *Rattus tunneyi *(Australia 4)	12800
*Bartonella *from *Isoodon macrouris *(Australia 9)	6400
*Bartonella *from *Uromys caudimaculatus *(Australia 14)	6400
*Bartonella *from *Melomys *(Australia 17)	12800
*Bartonella *from *Rattus cornuatus *(Australia 19)	6400
*Bartonella *from *Rattus leucopus *(Australia 20)	6400
*Azorhizobium caulinodans*	6400

### Immunofluorescence assay on 29 *Bartonella *strains array

**Figure 3:**
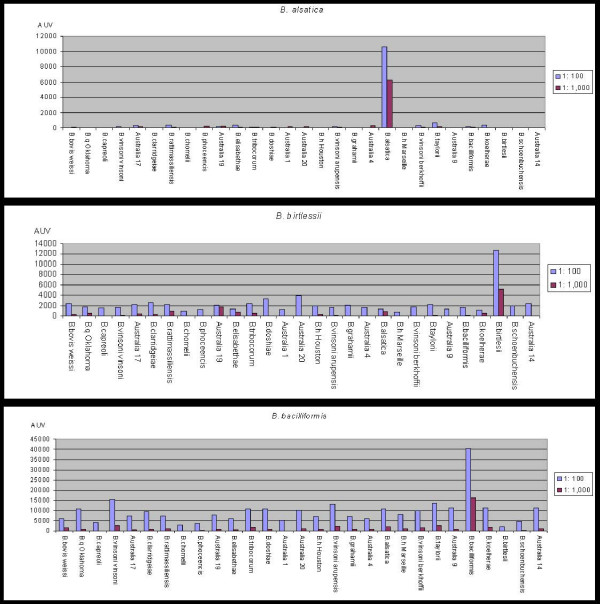
Low level of cross reaction for polyclonal antibodies against *B. alsatica*, *B. birtlesiii *and *B. bacilliformis*.

For all analysis, spots corresponding to *S. aureus *protein A and mouse IgG exhibited a strong value, indicating a good distribution of mouse serum sample and FITC conjugated antibodies. Spots of BSA and IgG goat AMCA showed very low values, demonstrating a slight background noise level of the assay. Initially, five sera from unimmunised mice were tested and we obtained mean values less than 432, 362 and 97 AUV, respectively, corresponding to the 1:100, 1:1,000 and 1:5,000 dilution assays (data not shown). We obtained a polyclonal sera specific for each of the strains in this study, however, the level of cross reactivity with other species varied greatly. Antibodies to *B. alsatica *and *B. birtlesii *showed strong and clear signals with very low levels of cross reactivity. *B. henselae *Marseille and *B. henselae *Houston generated polyclonal antibodies with a distinct microarray profile. For *B. henselae *Marseille, *B. taylori*, *B. birtlesii*, *B. bacilliformis*, *B. alsatica*, *B. quintana *Oklahoma, *B. henselae *Houston, *B. tribocorum*, *B. koehlerae*, *B. schoenbuchensis*, *B. doshiae*, *B. vinsoni arupensis*, *B. clarridgeiae, B. bovis*, *Bartonella *strains isolated from *Macropus giganteus *and from *Uromys caudimaculatus *polyclonal sera, we obtained a strong signal directed against the strain that was used for immunisation at the 1: 100 and 1: 1,000 dilutions. The values ranged from 4,600 AUV for *B. henselae *Marseille to 47,000 AUV for *B. tribocorum *at the 1: 100 dilution. In the case of the 1: 1,000 dilution assay, values ranged from 3,200 AUV for *B. henselae *Marseille to 27,000 AUV for *B. taylori *(data not shown). Among these polyclonal sera, some exhibited a low level of cross reactivity (Fig 3). For twelve other polyclonal sera, the 1: 1,1000 and 1: 5,000 dilutions were investigated due to the absence of a clear and strong specific signal and excessive cross-reaction at the 1: 100 dilution. The recorded values ranged from 2,500 to 14,000 AUV and from 500 to 9,000 AUV at two dilutions 1: 1,000 and 1: 5,000 for *B. phoceensis *and for the strain isolated from *Isoodon macrouris*, respectively. We concluded from this experiment that polyclonal sera against *B. quintana *Oklahoma, *B. henselae *Houston, *B. henselae *Marseille and *B. clarridgeiae *at the 1: 1,000 dilution can be used to perform serotyping.

### Bacterial strains from homeless persons and array for serotyping

**Figure 4:**
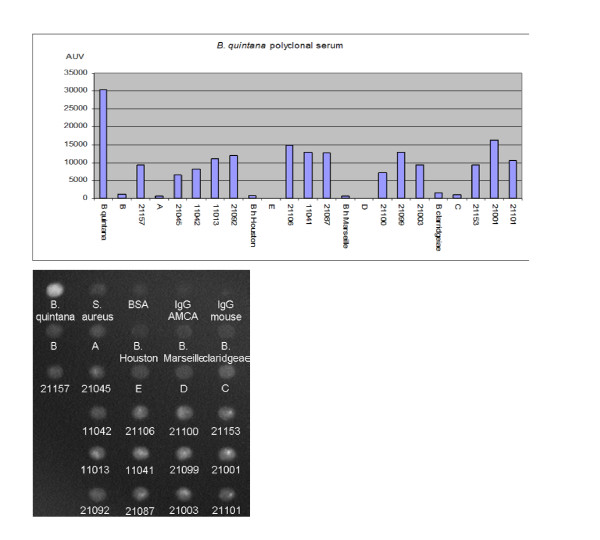
Graph representing the identification of bacteria from 14 homeless blood cultures anonymized as *B. quintana *by polyclonal mouse antibodies and photo representing direct data obtained after incubation and reading at 470 nm with monoclonal antibodies.

A total of 14 positive cultures accounting for 10.6% of the assays were detected from day 7 to day 14 after seeding blood culture medium on Columbia agar. Signals on the array were negative or very slight against the five blind test strains, but very strong against reference strains with the corresponding polyclonal serum. All the bacterial strains isolated by blood culturing from homeless persons gave a strong positive signal using polyclonal sera at the dilution indicated above. However, these signals were weaker than that of the *B. quintana *strain and we obtained comparable results with a *B. quintana *monoclonal antibody produce (Fig 4).

### Production of monoclonal antibodies

**Figure 5:**
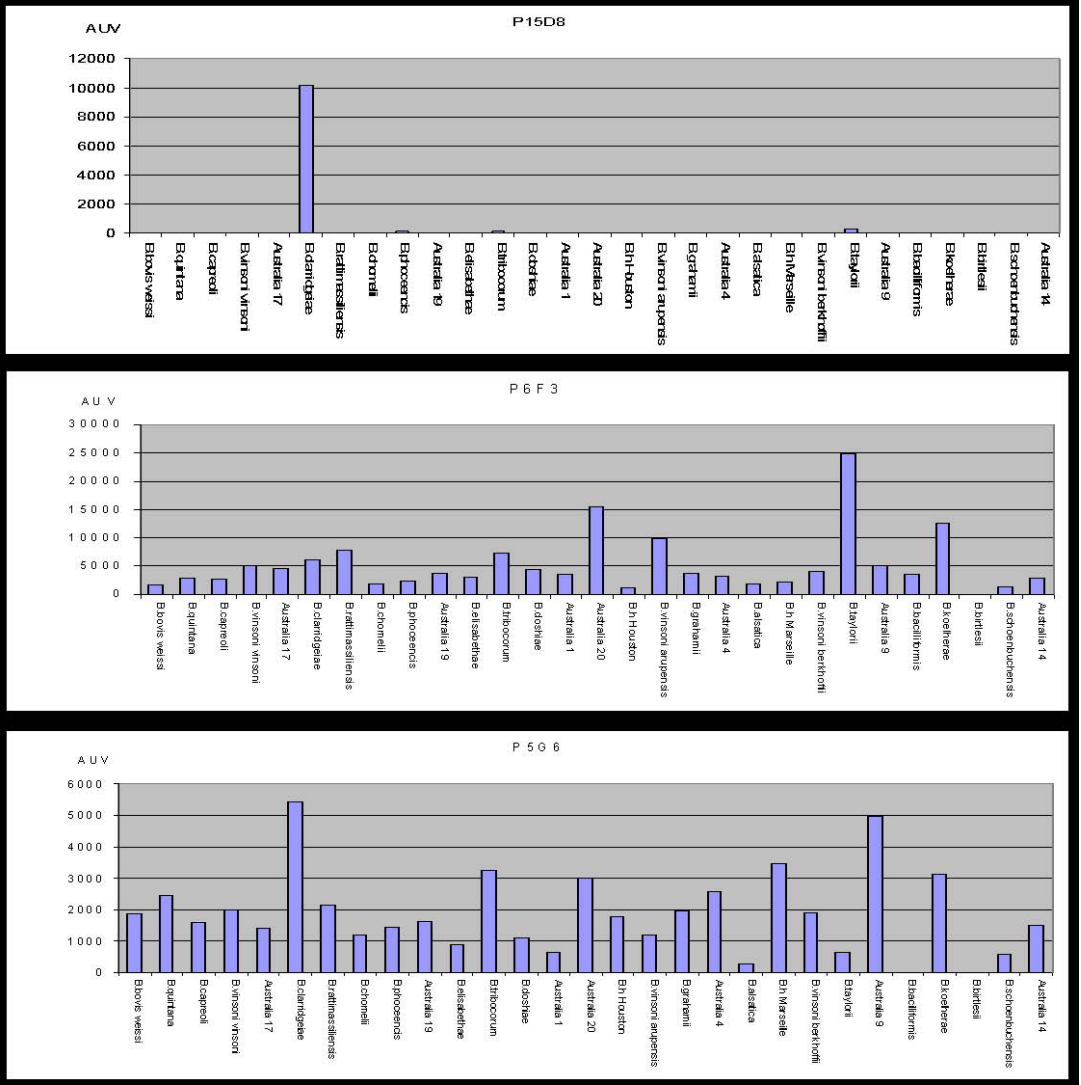
Cross reaction and specificity of monoclonal antibodies against the Gro EL protein.

In this study, we obtained a total of 712 hybridomas, of which 17 gave a positive signal on the indirect immunofluorescence assay. The investigated microarrays exhibited a negative signal against *B. birtlesii *for all the tested monoclonal antibodies. Nine monoclonal antibodies strongly reacted with all the other strains, whereas three monoclonal antibodies did not react against one of the strains, *B. bacilliformis*. Five monoclonal antibodies demonstrated a major affinity toward *B. clarridgeiae *but did not react against the other strains, especially *B. bacilliformis*, *B. henselae *Houston, *B. henselae *Marseille and *B. quintana *(Fig 5).

**Figure 1:**
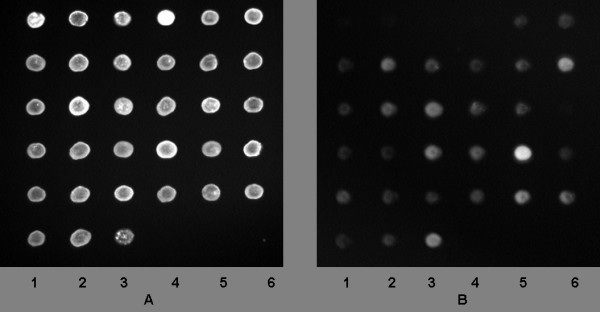
**A: Quality control of nanospotted antigens using 365 nm light exposure**. Lane 1 spotsfrom top to bottom: Mouse IgG; *B. bovis*; *B. quintana *Oklahoma; *B. capreoli*; *B. vinsonii vinsonii*; *Bartonella *from *Melomys*. Lane 2 spotsfrom top to bottom: *S. aureus *protein A; *B. clarridgeiae*; *B. rattimassiliensis*; *B. chomelii*, *B. phoceensis*; *Bartonella *from *Rattus cornuatus*. Lane 3 spotsfrom top to bottom: BSA; *B. elizabethae*; *B. tribocorum*; *B. doshiae*; *Bartonella *from *Macropus giganteus*; *Bartonella *from *Rattus leucopus*. Lane 4 spotsfrom top to bottom: IgG goat AMCA; *B. henselae *Houston; *B. vinsoni arupensis*; *B. grahami*; *Bartonella *from *Rattus tunei*. Lane 5 spots from top to bottom: *B. alsatica*; *B. henselae *Marseille; *B. vinsoni berkoffii*; *B. taylorii*; *Bartonella from Isoodonmacrouris*. Lane 6 spotsfrom top to bottom: *B. bacilliformis*; *B. koehlerae*; *B. birtlesii*; *B. schoenbuchensis*; *Bartonella from Uromys*. 1B: Same slide incubated with monoclonal antibody P8G6 and reading at 470 nm showing cross reaction and specificity of P8G6.

The monoclonal antibody P8G6 (Fig 1B) showed the best values to detect all *Bartonella*, except *B. birtlesii*. Detection values ranging from 0 to 527 AUV showed a low background noise for the four negative controls, whereas the detection values ranging from 2351 to 37133 AUV showed good detection for 28 strains. The nine monoclonal antibodies reacted differently the same strain, and there was no correlation between the values and the phylogeny of *bartonella *species based on *groEL *sequences (data not shown) [19].

## Discussion

The tools of choice for strain identification of *Bartonella spp*. are molecular tests, ever since the advent of multilocus sequence typing [20]. In fact, it is difficult to determine the *Bartonella *species that cause disease using polyclonal or monoclonal antibodies for classical immunofluorescence, because of extensive cross-reactivity and the removal of all detectable antibodies by cross-adsorption [21,22].

In the present work, we used an immunofluorescence assay in combination with microarray technology and automatic methods against twenty-nine different *Bartonella *strains at the same time. This serotyping technology for all strains simultaneously permit us to decrease cross reactivity and allows us to detect and identify each strain using polyclonal antibodies. Our method has also permitted the detection of new strains, such as *Bartonella *strains isolated from *Macropus giganteus *and from *Uromys caudimaculatus *using polyclonal antibodies. Our immunofluorescence-array assay exhibited sensitivity comparable to a classical single immunofluorescence assay, as we obtained a significant signal up to a 1:5,000 dilution against respective strains used for immunization. For some strains, specific signals were associated with cross-reactions. However, the intensity easily allowed the correct identification of the strain, except for *B. rattimassiliensis *polyclonal serum, which reacted with *B. tribocorum *as well. However, we easily discriminated between the strains, as the *B. tribocorum *polyclonal serum did not react significantly with *B. rattimassiliensis*. These signals were delivered without a previous cross-adsorption and without separate analysis. During the immunoassay, some problems may occur, such as a hook effect [23,24], due to the variation in the amount of spotted antigen that cannot be easily detected even by using different test controls. However, the problem can be avoided by testing several dilutions. Polyclonal serum can be unsuccessful due to possible variations in antigen recognition, selection of antibody secreting clones by the immune system control, or mutations that occur during the immune response [25,26]. The two *Bartonella *strains, *B. rattimassiliensis *and *B. tribocorum*, were isolated from rodents. Phylogenetic studies based on ITS, 16S rDNA, genes of four intracellular proteins and the *groEL *gene, have indicated close clusters for these two *Bartonella *strains, but also for all *B. vinsoni subsp*, for *B. phoceensis *and *B. alsatica*, and for *B. doshiae *and *B. taylorii *[27]. Our newly designed technique was successful in discriminating among all the strains in the last three clusters stated above, as well as strains responsible for human *Bartonella *diseases.

Using the second array, we identified known strains from homeless persons, such as *B. quintana*, confirming that urban trench fever is endemic in the homeless population. We demonstrated that 1 hour is sufficient to test four samples, suggesting that our new serological approach could be a practical tool for serotyping of isolated strains using specific polyclonal antisera and reference bacteria.

**Figure 6:**
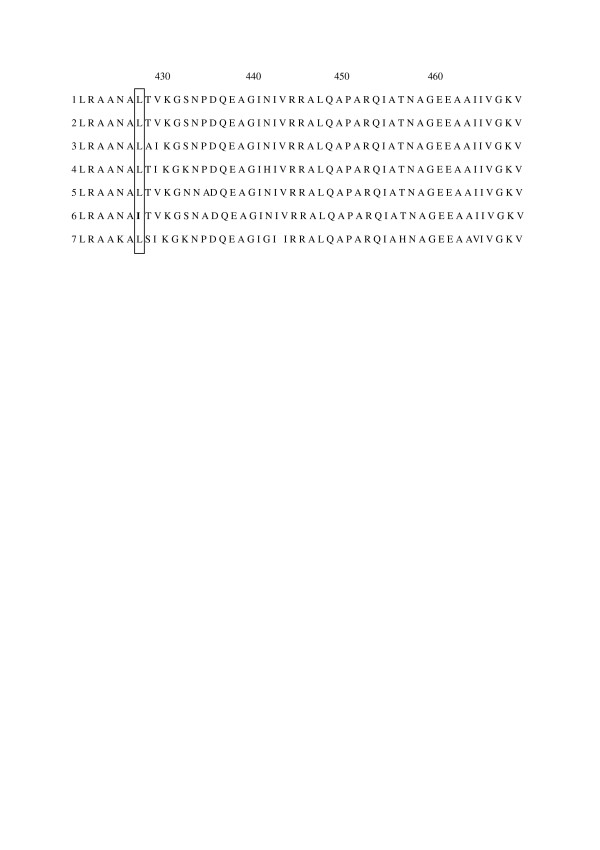
Sequence alignment of seven strains of *Bartonella*. 1: *B. taylorii*; 2: *B. henselae *Marseille, 3: *B. quintana *Oklahoma; 4: *B. clarridgeiaea*; 5: *B. bovis weissi*; 6: *B. birtlesii*; 7: *B. bacilliformis*, showing a mutation at position 427 for *B. birtlesii*.

We have also screened monoclonal antibodies produced *against B. claridgeae*, in order to find a monoclonal antibody that broadly reacts with the *Bartonella *species. The best result was obtained with the monoclonal antibodies against GroEL. Microarray revealed three categories, corresponding to preserved or variable epitopes of the GroEL protein in the genus *Bartonella*. This monoclonal antibody failed to react with *B. birtlesii *and the study has been repeated four times with the same negative results. However, polyclonal antibodies against *B. birtlesii *showed a good reactivity, excluding the defaults in the spotted antigen. The sequence alignments of seven strains of GroEL proteins (*B. bovis*; *B. quintana *Oklahoma; *B. clarridgeiae*; *B. henselae *Marseille; *B. taylorii*; *B. bacilliformis*; *B. birtlesii*;)[19](Fig 6), showed that a single mutation at position 427 (leucine in isoleucine) is specific to *B. birtlesii*. This mutation could explain the lack of reaction to *B. birtlesii *by the monoclonal antibodies and the important role of this amino acid in the conformation and recognition by the monoclonal antibody. New serological investigations with large-scale use of bacteria from the same species or the combined genus of the pathogens could be of interest in human health for the diagnosis of *Bartonella *diseases.

## Conclusion

We demonstrated that microarray of spotted bacteria can be a practical tool for serotyping of unidentified strains or species (and also for affinity determination) by polyclonal and monoclonal antibodies. This could be used in research and for identification of bacterial strains.

## Methods

### Preparation of antigens

For this experiment, twenty-nine *Bartonella *strains were used (Table 1). All strains were cultured on Columbia agar supplemented with 5% sheep blood (BioMerieux, Marcy l'Etoile, France) at 37°C using the Generbag system (BioMerieux, Marcy l'Etoile, France), except *B. bacilliformis*, for which cultivation was performed at 28°C. Bacterial colonies were scrapped off carefully from the agar plates and suspended in 10 ml phosphate buffer (pH 7.2, PBS) containing six glass beads (2 mm). Ethanol was added to obtain a final concentration of 75% and the bacterial suspensions were incubated overnight at 4°C. On the following day, each cell-suspension was washed twice with 20 ml sterile PBS and bacterial density was adjusted to 10^9^/ml after counting with Microcyte (BioDetect, Oslo, Norway), and then stored at 4°C. Several aliquots were removed from the cell-suspension for sterility controls, seeding two Columbia agar for protein quantification using Biorad protein assay (Biorad Laboratories, Ivry sur Seine, France) and for polyclonal antisera production. Sodium azide powder (Sigma Aldrich, Saint-Quentin Fallavier, France) was added to the rest remaining cell-suspension at a final concentration of 0.1% and stored at 4°C for spotting.

**Table 1:** Bacterial strains used for spotting

Species	Collection source^a^
*Bartonella alsatica*	CIP 105477 T
*Bartonella bacilliformis*	ATCC 35686
*Bartonella bovis*	CIP 106692 T
*Bartonella clarridgeiae*	ATCC 51734
*Bartonella elizabethae*	ATCC 49927
*Bartonella henselae *Houston	ATCC 49882
*Bartonella henselae *Marseille	CIP 104756 T
*Bartonella koehlerae*	ATCC 700693
*Bartonella quintana *Oklahoma	ATCC 51694
*Bartonella rattimassiliensis*	CIP 107705 T
*Bartonella tribochorum*	CIP105476 T
*Bartonella vinsonii arupensis*	ATCC 700727
*Bartonella vinsonii berkhoffii*	ATCC51672
*Bartonella birtlesii*	CIP 106294 T
*Bartonella capreoli*	CIP 106691 T
*Bartonella chomelii*	CIP 107869 T
*Bartonella doshiae*	NCTC 12862
*Bartonella grahamii*	NCTC 12860
*Bartonella taylorii*	NCTC12861
*Bartonella schoenbuchensis*	NCTC 13165T
*Bartonella vinsonii vinsonii*	ATCC VR-152
*Bartonella phoceensis*	CCUG 51222
*Bartonella *from *Macropus giganteus *(Australia 1)	CCUG 51999
*Bartonella *from *Rattus tunneyi *(Australia 4)	CCUG 52161
*Bartonella *from *Isoodon macrouris *(Australia 9)	CCUG 52002
*Bartonella *from *Uromys caudimaculatus *(Australia 14)	CCUG 52164
*Bartonella *from *Melomys *(Australia 17)	CCUG 52167
*Bartonella *from *Rattus cornuatus *(Australia 19)	CCUG 52169
*Bartonella *from *Rattus leucopus *(Australia 20)	CCUG 52174
*Azorhizobium caulinodans*	ATCC 43989

^a ^Abbreviations: ATCC, American Type Culture Collection, Manassas, Va; CIP, Collection de l'Institut Pasteur, Paris, France; NCTC, National Collection of Type cultures, Central Public Health Laboratory, London, United Kingdom; CCUG, Culture Collection University of Göteborg, Sweden.

### Production of polyclonal antisera

For each *Bartonella *strain, one six-week old female Balb/C mouse was inoculated intraperitoneally with a mixture of 10 μg bacterial protein, 400 μg aluminium hydroxide and 10 μg CpG, as previously described [28]. Two booster doses were given over a 14-day interval. Blood samples were drawn ten days after the last immunisation under isoflurane anaesthetic conditions (Abbott, Rungis, France). Sera were separated by centrifugation and stored at 4°C for serological tests. An indirect immunofluorescence assay was used to verify IgG response of immunised mice against their respective *Bartonella *strain. Antigens were deposited on slides with a pen nib. Slides were air-dried and fixed in methanol for 2 min at room temperature. Wells were overlaid with 30 μl of serum diluted in PBS containing 3% non-fat dry milk and incubated in a moist chamber at 37°C for 30 min. Afterwards, the slides were washed two times in PBS-0.5% Tween for 5 min each and rinsed for 4 min with desionized water. Slides were air-dried and bound-antibodies were detected with a FITC conjugated goat anti-mouse IgG (Immunotech, Marseille, France) diluted at 1: 400 in PBS containing 3% non-fat dry milk and 0.2% Evans blue (BioMerieux, Marcy l'Etoile, France). Slides were washed as described above and mounted with Fluoprep (BioMerieux, Marcy l'Etoile, France), and then examined under the Olympus BX-51 epifluorescence microscope at × 400 magnification. Sera from five healthy mice were used as a negative control.

### Immunofluorecence assay on 29 *Bartonella *strains array

We developed an automatic and miniaturized technology of an immunofluorescence assay, derived from classical immunofluorescence. Antigens were spotted by using a micoarray spotter robot on glass slides to simultaneously analyse the immunological response against all the strains of bacteria. The spotter deposited 1 nl (100 at 200 μm of diameter) onto each spot separated by 500 μm. We used goat IgG (Sigma-Aldrich, Saint Quentin Fallavier, France) to stick and increase the thickness of antigens to the glass slides and to control spotting quality. Spotted slides were fixed in ethanol for 10 min and air-dried as in classical immunofluorescence. Antigenic spots were identified at 395 nm by staining the goat IgG with a fluorophore, 6-((7-amino-4-methylcoumarin-3-acetyl) amino) hexanoic acid (AMCA) (Interchim, Montluçon, France). Five milligram portions of goat IgG were labelled with AMCA by suspending IgG in 1 ml PBS (pH 8.0) and then mixed with 40 μl AMCA 0.5% for 30 min in the dark. AMCA labelled goat IgG were purified by washing using YM-30 Microcon centrifugal filters (Millipore, Saint-Quentin en Yvelines, France) and stored at -20°C. Specific proteins (1 mg/ml) used as controls were simultaneously distributed as separate spots to validate each immunofluorescence assay step. Protein A from *S. aureus *(Sigma Aldrich, Saint Quentin Fallavier, France)[29] was used to verify the serum by unspecific protein A binding, whereas mouse IgG (Sigma Aldrich, Saint Quentin Fallavier, France) was used to monitor secondary antibody distribution. Bovine serum albumin (Sigma Aldrich, Saint Quentin Fallavier, France) and goat IgG AMCA were assayed to evaluate background noise. Thirty-five microlitres of each bacterial or protein suspension were mixed with 15 μl of labelled goat IgG, and distributed into 96-well plates. 35 to 50 pl of each antigen was deposited using an Affymetrix 427 Arrayer (MWG Biotech, Courtaboeuf, france) on SuperFrost glass slides (Menzel-Glasser, Braunschweig, Germany) previously degreased in a deionised water bath with a Leo 50 ultrasonic cleaner (FisherScientific, Illkirch, France) for 5 min, followed by 10 min immersion in acetone/ethanol (50/50 v-v). Spot quality was verified by observing the slides under a 365 nm-wavelength light (Fig 1) and slides with perfect circular spots without smears were stored at room temperature in the dark.

The immunofluorescence assay was performed on spotted slides at room temperature in an automated incubator, with which four different sera could be run in parallel (Inodiag, La Ciotat, France). Polyclonal sera were diluted in PBS containing 0.5% sodium azide and 5% goat serum. Forty-five microlitres of the diluted sera were distributed into circular steel-chambers. Each spotted slide was clamped over each chamber, which allowed sealing and contact between the slides and deposits. All of the necessary steps, such as the continuous distribution of reagents, including secondary antibody addition, washing and drying steps, were automatically executed in the chamber. After 20 min of incubation, the diluted sample was removed and the slide was washed for 3 min by flooding with a solution of PBS-0.5% sodium azide-10% goat serum. Distribution of secondary FITC-conjugated antibody (Beckman Coulter, Marseille, France) was performed in PBS-0.5% sodium azide-10% goat IgG solution at 1: 150 dilution for 20 min, followed by a washing step as described above and was then rinsed with deionised water for 1 min and air-dried for 5 min. At the end of the incubation, slides were examined with a fluorescent camera analyser (Inodiag, La Ciotat, France) with a successive exposure to 365 nm and 480 nm light during 0.2 ms each, and the arbitrary unit values (AUV) were automatically allotted for each spot using data processing Analarray 4.4-1 (Inodiag, La Ciotat, France) in the following manner: the first reading at 365 nm determined the area of each spot and verified the presence of all antigens. The view obtained after reading at 470 nm was superimposed with that obtained at 365 nm and fluorescence of the defined area was converted into a grey scale pixel image. Values were then calculated on the defined grey-coloured pixels. Analysis was rejected if some spots were not detected after reading at 365 nm or if the fluorescence level of protein control spots at 470 nm was too low. Four slides can be simultaneously treated on the same incubator with a gap of one hour and the reading and interpreting of one slide could be done in 1 minute. For this experiment, we subtracted BSA spot values from all the other spot values obtained. All measurements were carried out in triplicate with a standard deviation +/- 500 AUV and the average values were considered.

### Bacterial strains from homeless and array for serotyping

*Bartonella *strains were obtained for the study from two shelters visited in Marseilles by a medical team, as in previously described studies [30]. Isolated bacteria were expanded and submitted to the same treatment as *Bartonella *reference strains. A new array for serotyping was constituted with these strains, four *Bartonella *reference strains (*B. quintana*, *B. clarridgeiae*, *B. henselae *Houston and *B. henselae *Marseille), five bacteria strains as blind test control (A: *B. alsatica*; B: *B. vinsoni berkhoffii*; C: *B. koehlerae*; D: *A. caulinodans*; E: *B. elizabethae*) and four protein controls (mouse IgG, BSA, protein A and goat IgG). *B. quintana *monoclonal antibody was used to compare with polyclonal sera. This monoclonal antibody was produce in our lab for this study with a titer at 6400 and use at dilution 1/3.

### Monoclonal antibody production

**Figure 2:**
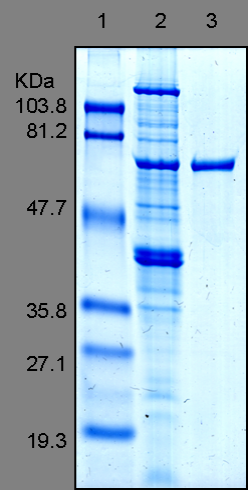
Blue stained SDS-PAGE of the purified GroEL protein (Lane 3) and the *Bartonella clarridgeiae *whole cell-extract (lane 2). Standard molecular weight ladders (Lane 1) are indicated on the left.

In order to obtain *Bartonella *monoclonal antibodies identifying all *Bartonella*, we previously tested the different *Bartonella *strains with mouse polyclonal antibodies by western blot assay. We found that *Bartonella clarridgeiae *antisera can react with the GroEL protein from all *Bartonella *and we selected this antigen to produce broad spectrum *Bartonella *monoclonal antibodies (data not shown). A cell pellet of *Bartonella clarridgeiae *(100 μg) was prepared as previously described, suspended in Laemmli buffer and heated for 5 min at 95°C. The insoluble fraction was removed by centrifugation at 10,000 × *g *for 10 min. Soluble proteins were resolved by 10% SDS-PAGE, followed by Coomassie blue staining (0.2% Methanol, 0.5% TCA, 0.1% (w/v) Coomassie Brilliant Blue G). The major GroEL protein was extracted from the polyacrylamide gel by the electroelution method using the ElutaTube™ Protein Extraction Kit (Euromedex, Mundolsheim, France) according to the manufacturer's protocol (Fig 2). Three six week old female mice were intraperitoneally inoculated with 1 μg *gro EL *protein as described above. Four days after the last booster dose, spleen cells were submitted to polyethylene glycol fusion (Roche Diagnostic, Meylan, France) with X63 Ag 8.653 myeloma cells. Fused cells were seeded in a hypoxanthine-aminopterine-thymidine selective medium (Invitrogen, Cergy-Pontoise, France) and hybridoma supernatants were screened by immunofluorescence assay using *B. clarridgeiae *strain as mentioned above. Positive hybridomas were subcloned by the limiting dilution method.

## Competing interests

Didier Raoult is a cofounder of INODIAG, a startup localized in LA CIOTAT France. The other authors declare no competing interests.

## Authors' contributions

CN carried out the production of antibodies, the preparation of antigens, the incubation and analysis of microarray and participated in drafting the manuscript. CB participated in the preparation of antigens, the concept and design of the microarray and the drafting of the manuscript. DR conceived of the study, and participated in its design and coordination and helped to draft the manuscript. All authors read and approved the final manuscript.
